# Forward Modeling of Fluctuating Dietary ^13^C Signals to Validate ^13^C Turnover Models of Milk and Milk Components from a Diet-Switch Experiment

**DOI:** 10.1371/journal.pone.0085235

**Published:** 2013-12-31

**Authors:** Alexander Braun, Stephan Schneider, Karl Auerswald, Gerhard Bellof, Hans Schnyder

**Affiliations:** 1 Lehrstuhl für Grünlandlehre, Department of Plant Science, Technische Universität München, Freising, Germany; 2 Fachgebiet Tierernährung, Fakultät Land- und Ernährungswirtschaft, Hochschule Weihenstephan-Triesdorf, Freising, Germany; Paris Institute of Technology for Life, Food and Environmental Sciences, France

## Abstract

Isotopic variation of food stuffs propagates through trophic systems. But, this variation is dampened in each trophic step, due to buffering effects of metabolic and storage pools. Thus, understanding of isotopic variation in trophic systems requires knowledge of isotopic turnover. In animals, turnover is usually quantified in diet-switch experiments in controlled conditions. Such experiments usually involve changes in diet chemical composition, which may affect turnover. Furthermore, it is uncertain if diet-switch based turnover models are applicable under conditions with randomly fluctuating dietary input signals. Here, we investigate if turnover information derived from diet-switch experiments with dairy cows can predict the isotopic composition of metabolic products (milk, milk components and feces) under natural fluctuations of dietary isotope and chemical composition. First, a diet-switch from a C_3_-grass/maize diet to a pure C_3_-grass diet was used to quantify carbon turnover in whole milk, lactose, casein, milk fat and feces. Data were analyzed with a compartmental mixed effects model, which allowed for multiple pools and intra-population variability, and included a delay between feed ingestion and first tracer appearance in outputs. The delay for milk components and whole milk was ∼12 h, and that of feces ∼20 h. The half-life (t_½_) for carbon in the feces was 9 h, while lactose, casein and milk fat had a t_½_ of 10, 18 and 19 h. The ^13^C kinetics of whole milk revealed two pools, a fast pool with a t_½_ of 10 h (likely representing lactose), and a slower pool with a t_½_ of 21 h (likely including casein and milk fat). The diet-switch based turnover information provided a precise prediction (RMSE ∼0.2 ‰) of the natural ^13^C fluctuations in outputs during a 30 days-long period when cows ingested a pure C3 grass with naturally fluctuating isotope composition.

## Introduction

Isotopic variation in food stuffs propagates through trophic systems generating isotopic tags in organisms at each trophic level [1]. Metabolically active tissues steadily degrade and renew and thus integrate dietary isotopic signals [2]. Propagation of natural dietary isotopic variation was found in wildlife and livestock species, and also in humans [3], [4], [5]. For carbon, such dietary variation may result from differences in carbon isotopic discrimination between plants with different photosynthetic mechanisms and variation within each mechanism resulting from species-specific morpho-physiological properties and responses to environmental drivers [6], [7]. Moreover, post-photosynthetic fractionation phenomena can generate variation in carbon isotope composition (δ^13^C) of chemical compounds and plant parts [8].

A delay can be expected from feed intake to first appearance of dietary isotopic signals in tissues [9], due to ingestion and passage time. The rate of incorporation of “new” diet isotopes and simultaneous loss of “old” isotopes in tissues of consumers is then driven by metabolic processes, such as cycling and storage, and can be comprehensively quantified as isotopic turnover. Turnover implies that isotopic variation in consumer tissues is attenuated, meaning that the isotopic composition of the tissue fluctuates less than that of the diet [10]. The degree of attenuation is inversely related to turnover rate; thus, attenuation is weak if turnover is fast and strong if turnover is slow.

Turnover of tissues in different animal species, here assessed as half-lives, range from <1 d [11] to >100 d [12], [13]. Half-lives are modified by endogenous and exogenous factors: in particular, half-lives differ between species (e.g. cattle and rats, [12], [14]), organs/tissues (e.g. liver and blood, [15]) and between chemical compounds (e.g. carbohydrates and proteins, [16]). For example, the carbon isotopic half-lives vary between muscle tissue of cattle and rats by 120 d, because isotopic turnover rate declines with body mass following the −¼ power [17]. Half-lives also vary within the same species between different tissues, e.g. liver and muscle of *Rattus norvegicus* by 30 d [14], likely as a result of differences in protein turnover [17]. Moreover, half-lives vary with growth [18], temperature [19], diet composition [11], [20], [21], sex and age [22]. In particular, the carbon isotopic half-life of blood in nectarivorous bats varied by 60% following a change in the diet [21].

Half-lives are assessed in isotopic or diet switch experiments [12], [23], [24]. Such experiments involve a systematic shift of the isotopic composition of the diet, recording of the time course of the isotopic composition in the outputs of interest and compartmental modelling of the tracer data. Compartmental modelling has frequently been used to investigate the kinetic properties of metabolic and storage pools [17], [25], because it allows inferring the number of kinetically different pools in a metabolic system, and their specific pool half-lives and relative sizes. In diet switch experiments, animal and environmental conditions as well as diets are usually strictly controlled in order to realize close-to-steady-state conditions, except for the isotopic composition of the diet that is altered at the time of the switch. In principle, the steady-state conditions are violated if the isotopic switch involves a switch in diet chemical composition, as is frequently the case (this study, [12], [21], [23], [26], [27], [28]). Such non-steady state conditions, in combination with fluctuating environmental conditions, question the validity of diet-switch based turnover parameters and data. It is a difficult – if not impossible – task to maintain the chemical composition of the diet while changing its isotopic composition, particularly in studies with large herbivores, as is also the case in the present study. For instance, replacement of C_3_ grasses by C_4_ grasses, including maize, generates a strong shift in δ^13^C, but it also means a shift from fructan-dominated to fructan-free non-structural carbohydrate contents [29] and a shift in feed tissue structure and anatomy (e.g. Krantz anatomy [30], [31]). Switches between grasses and legumes usually involve changes in protein content, tissue composition, and cell wall structure and chemistry [30]. Yet, essentially all of our knowledge on isotope turnover in large mammals, particularly herbivores, has been derived from diet-switch experiments of the above type. For this reason, there is an interest in the validation of diet-switch based turnover information under conditions differing from estimation. In particular, it is of interest to know if such models – derived under near-constant conditions – can predict the propagation of natural fluctuations in diet isotope composition to the products/outputs like milk. Fluctuations in the composition of feed are likely to be associated with changes in digestibility. Feces should hence show opposite behaviour to metabolic products, as a large proportion of the feces does not pass the gastrointestinal barrier to become part of the inner body, particularly the systemic circulation, but moves through the digestive tract as undigested residue [32]. Comparing metabolic products with feces thus increases the sensitivity for validating turnover models.

In this study with dairy cows, we investigated if diet-switch based turnover information (i.e. models and their parameters) allows the predicting of isotopic composition of metabolic outputs (milk, milk components and feces) at different dietary conditions that showed naturally fluctuating δ^13^C. To this end, we assessed the changes of δ^13^C in whole milk, lactose, casein, milk fat and feces in a diet-switch experiment. The data were analyzed with a compartmental mixed effects model, which allowed for multiple pools and intra-population variability. The model also included a delay between ingestion and first appearance of tracer in whole milk, milk components and feces. Then, we validated the model of the whole milk by back calculating whole milk from its components, fitting a model to the back calculated whole milk and comparing this model with the model of the original milk. The back calculation was done by adding the measured values of all three components according to their relative contribution to the carbon content of the whole milk. Finally, we assessed the performance of the models in predicting the δ^13^C of outputs at different dietary conditions that showed naturally fluctuating δ^13^C.

## Materials and Methods

### Ethics Statement

Since the experiment involved typical agricultural practices of animal keeping, no specific regulations/laws applicable for experiments involving vertebrates had to be followed. The animals were kept and fed at the farm of the University of Applied Sciences Weihenstephan, under the authority of the government of the Bavarian administrative district Oberbayern. All animals were healthy and remained at the farm after the conclusion of the experiment. Blood was drawn by an attending veterinarian for monitoring of health and well-being; the samples for this study were taken by SS.

### Animals, Feeding and Labelling

The diet switch experiment was performed with eight Simmental cows selected from a larger herd to yield a subsample with similar age, milk production and phase of lactation. The cows were 4.2 years old (SD 0.4 yr; SD indicating the standard deviation), produced 22.6 L d^−1^ (SD 1.6 L d^−1^) of milk and were in mid lactation (162 d in milk SD 14 d). However, the cows differed in live weight by up to 153 kg (mean 640 kg SD 56 kg, minimum 550 kg, maximum 703 kg). Each cow’s weight stayed approximately constant (SD 3%) during the experiment.

**Figure 1:**
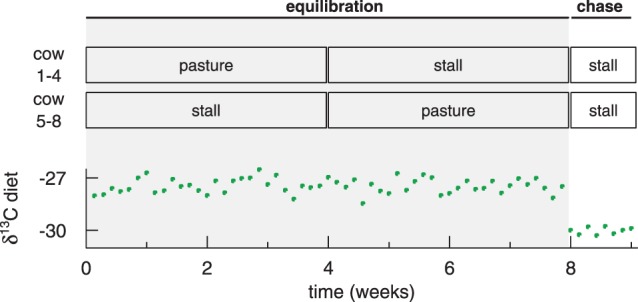
The experimental setup. The experiment comprised an isotopic equilibration period lasting eight weeks and a chase period lasting ten days with different dietary δ^13^C. The equilibration period was conducted as a cross-over experiment during which two groups of cows were switched between stall and pasture.

Prior to the experiment, the cows grazed for six weeks on a pasture, where grass (C_3_) was the only feed source. From the start of the experiment, the cows were fed a mixed diet consisting of fresh pasture grass supplemented with maize meal (C_4_) for eight weeks (this period was termed the ‘isotopic equilibration period’). The pasture grass was fed *ad libitum*. The maize meal was given at a constant rate (dry matter: 1.7 kg cow^−1^ d^−1^). The digestibility of the maize meal and pasture grass was high and near identical (80% and 77%, respectively, [33]). Since also the grass intake (dry matter: 16.2 kg d^−1^, SD 1.7 kg d^−1^) was similar between cows, the δ^13^C of the whole diet was very similar between cows (δ^13^C −27.40 ‰, SD 0.45 ‰). The equilibration period was designed as a cross-over experiment to examine whether the results obtained in the stall can also be applied for grazing (Fig. 1). During half of the period, four cows were kept in the stall, where grass intake could be measured (mass and isotopic composition), while the other four were kept on pasture except for the milkings, during which the cows also received the maize supplement. After half of the equilibration period, the groups were switched so that the cows kept on pasture were moved to the stall and *vice versa*.

An all-day pasture (3.0 ha, semi-natural grassland) with continuous stocking at 2.8 cow ha^−1^ was chosen, because it restricted selection during grazing and assured similarity of the grass fed on pasture and in stall. The target sward height for the all-day pasture was 6–7 cm and controlled daily (ca 150 measurements with a rising-plate meter from Ashgrove, RD 10, New Zealand) to prevent an ontogentic change in grass quality. To assure a similar diet for both groups of animals, grass for the stall group was harvested with a fodder harvester (Hege 212B, Waldenburg, Germany) between the grazing cows (fresh matter harvest approximately 450 kg d^−1^). The grass was cut to a stubble height of 3 cm by assuming the ‘take half’ rule for mean bite depth [34], [35]. Cutting was also used to maintain the target sward height when growing conditions allowed for more growth than consumed by the cattle. For more details see [33].

After eight weeks of equilibration, the maize supplement was eliminated from the diet, thus switching to a pure grass diet for the following ten days (‘chase period’).The equilibration period was six times longer than the chase period. This long equilibration procedure aimed at an isotopic equilibration of all body-pools that might contribute to the biosynthesis of milk components.

### Sampling and Isotope Analysis

We sampled the fresh grass and maize meal twice a day for each cow during the whole experiment. Grass samples were taken from the pasture grass that was harvested daily for feeding the cows in the stall. Further, we sampled and analyzed milk, milk components and feces for each cow four times a week (Monday evening, Tuesday morning, Thursday evening and Friday morning) during the equilibration period. The sampling interval was shortened during the chase period to quantify the isotopic turnover with greater accuracy. Here, samples were collected twice daily (at each milking).

Feces and diet samples were oven dried (60°C, 48 h) and milled. A subsample of whole milk was homogenized and freeze-dried. Another subsample was separated into milk fat, casein and lactose. First, milk fat was collected as the supernatant following centrifugation for 12 min at 2500 g. The casein was then precipitated by acidification to pH 4.3 with 10% HCl and subsequent centrifugation (30 min at 2500 g). The residue (whey), containing lactose and whey proteins, was heated to 80°C and filtered to remove the whey proteins and deliver lactose.

Aliquots of samples (dry matter: 0.7 mg, SD 0.05 mg; liquids: 5 µl, SD 0.1 µl) were transferred to tin cups and were combusted in an elemental analyzer (NA 1110, Carlo Erba, Milan, Italy) interfaced (Conflo III, Finnigan MAT, Bremen, Germany) to an isotope ratio mass spectrometer (Delta Plus, Finnigan MAT). Each sample was measured against a laboratory working standard CO_2_ gas, which had previously been calibrated against a secondary isotope standard (IAEA-CH_6_). Carbon isotope composition is presented as isotope ratio relative to the isotope ratio in the Vienna Pee Dee Belemnite standard and is expressed in parts per thousand (‰). SD of analytical repeats was 0.19 ‰ for whole milk, 0.11 ‰ for casein, 0.13 ‰ for lactose, 0.14 ‰ for milk fat and 0.12 ‰ for feces. After each tenth sample an internal lab standard with similar carbon ratio was run as a control.

### Statistical Analysis

We accounted for a delay between diet (input) switch and first output reaction and performed compartmental mixed effects modelling with the diet switch data. Compartmental modelling disentangles kinetically different pools contributing to an output [17]. Mixed effects models allow a statistically rigorous investigation of intra-population variability [36]. This approach comprised the following three steps:

First, we separated samples belonging to the delay period from samples belonging to the chase period. Samples were allocated to either period by stepwise testing sample t_0_ (time at diet switch) against sample t_e_ (time e hours after diet switch) by applying the Wilcoxon matched pairs test. The first sample at t_e_ that was significantly different from sample t_0_ was considered to be part of the chase period.

Second, the time course of the isotopic composition of the output (*δ^13^C_output_*) during the chase period was analyzed by a compartmental mixed effects model. For compartmental modelling, multi-exponential decay functions
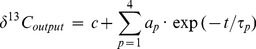
(1)were fitted to the data. The asymptotic base-line, which corresponds to the δ^13^C resulting from the pure pasture grass diet, is represented by *c*. The index number of exponential terms (pools) is *p*. Parameter *a* depends on the initial deviation from *c*, and the relative contribution of the individual pools when the delay is subtracted from time *t*. The parameter *τ_p_* is the mean residence time from which the half-life *t*_½_ of pool *p* can be derived as *τ_p_* • ln(2). The corresponding 95% confidence intervals were calculated as *t*_½_ ± SE • *T*_a_, where SE is the standard error of estimated *t*_½_ and *T*_a_ is the 97.5 percentile of the Student’s t-distribution with n-1 degrees of freedom. The equation parameters are directly valid for independent pools. However, several layouts of interacting pools can be fitted with this equation, but the interpretation of the parameters is then not straightforward anymore [37]. A pool may appear slow despite its fast turnover if it is not directly connected to the input and output. A decision on which layout of pools likely is valid cannot be made on statistical criteria due to the statistically similar performance of different layouts. It must be based on the physiological properties of the system [38]. We will show that independent outputs are the most likely interpretation in our case.

Models with up to four exponential terms were calculated. The number of exponential terms can be considered as the number of metabolic pools with different kinetic characteristics. Pools that differ biochemically but follow kinetics that cannot be resolved statistically are assigned to one pool, despite their biochemically contrasting nature. The mixed effects model technique accounted for fixed (time) and random (cows) factors in the compartmental models (for a detailed explanation of fixed and random factors see [39]). In particular, it allowed to decide on statistical criteria, whether the parameters *a*, *τ_p_* and *c* were identical among all cows, or whether individual parameter estimates were advantageous. This permitted the simultaneous analysis of all eight cows, but avoided pseudoreplication, since repeated measures were obtained from the same individuals. Akaike’s Information Criterion (AIC, [40]) was then used to select the model best supported by the data among the models with different numbers of exponential terms and models with different random and fixed factors.

Third, the delay period was estimated by calculating the time at the intersection of the accepted model with the mean of δ^13^C values of the previously estimated delay period. For comparison with other studies that ignored delays, we calculated a gross half-life. This represents the time after the diet switch (including the delay) at which half of the total output shift can be expected. In contrast to half-lives of individual pools, it applies only for the first half-life period but not later on because the delay would not be effective during the following half-life periods as long as there is no further diet switch.

To validate the milk component separation and measurement, we back calculated milk δ^13^C from its components casein, lactose and milk fat for every milk sample accounting for the relative contribution of each component to the total carbon content of milk. The deviation between back calculated and original whole milk as estimated by the root mean squared error (RMSE) was negligible (0.09‰ for the mean values of the cows; the average SD was 0.4 ‰). This indicated that artefacts during separation and isotope analysis were likely small. Hence, original and back calculated whole milk had similar pool kinetics that were statistically not different (Table 1).

**Table 1:** Compartmental model parameters for the outputs feces, milk components, whole milk and back calculated whole milk that was calculated from the milk components: pool number, delay, pool half-life and gross half-life resulting from the delay and the pool half-lives; 95% confidence intervals of the mean are given in parenthesis.

Output	Pool	Delay (h)	Pool half-lives (h)	Gross half-lives (h)
Feces	1	20 (19–22)	9 (6–12)	29
Lactose	1	12 (10–13)	10 (8–12)	22
Casein	1	12 (10–14)	18 (15–21)	30
Milk fat	1	12 (10–14)	19 (17–21)	31
Whole milk	1	12 (10–15)	10 (8–12)	28
	2		21 (18–24)	
Back calculated whole milk	1	12 (10–14)	11 (8–14)	28
	2		22 (18–26)	

The applicability of the diet-switch based turnover models and their parameters to fluctuating diet isotope composition was tested with data from the equilibration period. This took advantage of the naturally occurring short-term fluctuations in the δ^13^C of the grass component during the equilibration period. We calculated each output value at time “now” (δ^13^C_ouput;now_) from previous input values starting six half-lives (referring to the maximum half-life of a multi-pool system) and the delay period before “now” according to
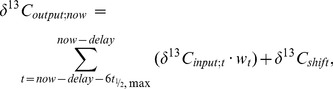
(2)where *δ^13^C_input;t_* is the input (dietary) isotopic signature at time *t*, and *w_t_* is the weighting factor at time *t* within the considered time period from time “now – delay –6*t*_½,max_” until “now – delay”. We neglected inputs of times greater than six half-lives of the slowest pool (*t*_½,max_), because they contribute <0.01%. The parameter *δ^13^C_shift_* indicates the trophic shift between input and output.

The weighting factor *w_t_* results from Eqn 1 as
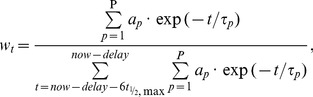
(3)where the numerator is the value predicted by the compartmental model (Eqn 1, the constant offset parameter c can be neglected) for time *t*, and the denominator is the sum of values of the compartmental model during six half-lives for which inputs were considered. The parameter P is the number of previously estimated pools. For the measured output, the 95% prediction intervals were calculated as *δ^13^C_output_* ± SD • *T*_a_ (where *T*_a_ is again the 97.5 percentile of the Student’s t-distribution) to examine whether the model output falls within the predicted range.

For the cows grazing on the pasture the same calculations were applied, except that the unknown grass intake was replaced by the average grass intake of the respective cow as measured during its stall period, while the same isotopic composition was assumed as that measured for the same day for grass fed in stall. Hence, only maize intake and its isotopic composition were known. The predicted δ^13^C of the outputs of the equilibration period for both stall and pasture cows were then compared with the independently measured δ^13^C values.

The shift *ε* in δ^13^C of outputs following the diet switch was calculated as
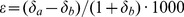
(4)where the δ^13^C before the switch (*δ_b_*) was given by the δ^13^C of the delay period, and the δ^13^C after the switch (*δ_a_*) was given by the fitted parameter *c* of Eqn 1, since *c* represents δ^13^C after complete turnover. All analysis was done in R 2.12.1 [41] with the auxiliary package nlme [36].

## Results

### Isotopic Characterization of Inputs and Outputs

The δ^13^C of the diet (fresh pasture grass and maize meal) and of whole milk, milk components (lactose, casein, milk fat) and feces collected from eight cows over nine weeks (including the equilibration and the chase period) provided a total of 2013 samples and isotope analyses. This included 133 samples of inputs (diet) and 1880 samples of outputs (milk, milk components or feces). The output samples divide into 47 samples per output per cow, with 32 samples from the equilibration period and 15 samples from the chase period.

The δ^13^C of inputs and outputs showed no significant trend over the equilibration period. The δ^13^C of inputs also showed no significant trend over the chase period. Nevertheless, naturally occurring short-term fluctuations were apparent in the inputs at both periods (SD 0.45 ‰ in equilibration and 0.38 ‰ in chase period) and also in the outputs (0.15 ‰) over the equilibration period. During the equilibration period, δ^13^C was −29.06 ‰ (SD 0.43 ‰) for grass and −12.14 ‰ (SD 0.21 ‰) for maize meal. Given the contribution of grass (90.2%, SD 0.6%) and maize meal (9.8%, SD 0.6%) to the diet, the δ^13^C of the input was −27.40 ‰ (SD 0.45 ‰). The carbon and nitrogen contents of the input during the equilibration period were 45.4% (SD 0.6%) and 3.2% (SD±0.4%) respectively. Milk fat (−30.23 ‰, SD 0.67 ‰), feces (−29.51 ‰, SD 0.52 ‰) and whole milk (−28.02 ‰, SD 0.48 ‰) were isotopically depleted in ^13^C compared to the input, whereas lactose (−26.94 ‰, SD 0.38 ‰) and casein (−25.39 ‰, SD 0.67 ‰) were isotopically enriched in ^13^C compared to the input. The δ^13^C of the input changed to −30.04 ‰ (SD 0.38 ‰) with the start of the chase period, creating an input switch of 2.7 ‰. The carbon and nitrogen contents of the input during the chase period were 41.8% (SD 0.6%) and 4.0% (SD 0.4%), respectively.

### Compartmental Mixed Effects Modelling

The δ^13^C of outputs did not change immediately after the input switch but exhibited a delay. Usually the first two measurements, one at the switch, the other at 12 h later, did not differ significantly from the mean δ^13^C of the equilibration period. Thus, these samples were included in the delay period. Delay periods, calculated as the time of intersection between the turnover model with the mean δ^13^C during the equilibration, were relatively short for casein, lactose, milk fat, whole milk and back calculated milk (about 12 h), and longer for feces (20 h, Table 1).

**Figure 2:**
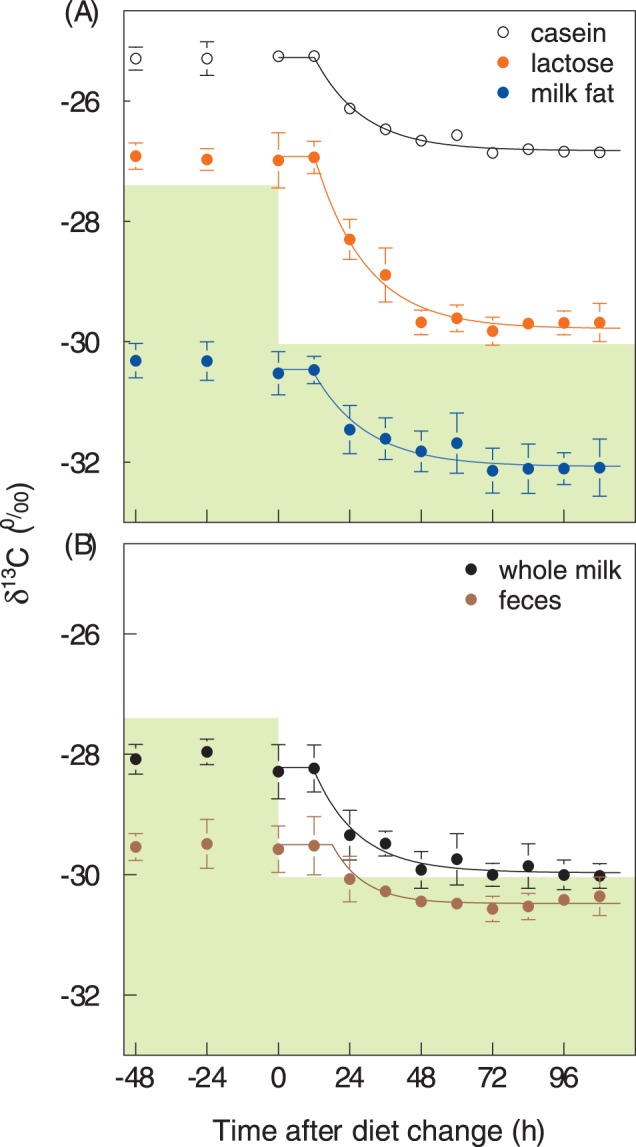
Isotopic time course of the outputs. Time course of δ^13^C in milk components (panel A) and in whole milk and feces (panel B) for a switch from a mixed diet to a pure grass diet. The mean diet δ^13^C is shown in green. Error bars denote the 95% confidence intervals of the mean (n = 8 for each data point). Solid lines denote the turnover models, including a delay.

After the delay, the δ^13^C of all outputs changed rapidly to reach a near-constant value after two to three days. The δ^13^C of the last four measurements could no longer be distinguished, indicating that the changes within these two-day periods were smaller than the confidence interval based on the experimental error and the number of replicates (Fig. 2). The shift in δ^13^C following the diet switch (−2.7 ‰) differed among outputs. It was largest in lactose (−2.9 ‰, SD 0.1 ‰) and smaller in whole milk (−2.1 ‰, SD 0.1 ‰), milk fat (−2.0 ‰, SD 0.3 ‰), casein (−1.6 ‰, SD 0.1 ‰) and feces (−1.1 ‰, SD 0.1 ‰).

The AIC statistics supported only one pool for each milk component and for the feces (Table 1). Half-lives were short for feces and lactose (9 h and 10 h) and longer for casein and milk fat (18 h and 19 h). Conversely, whole milk was best fitted with a two-pool model including a fast pool (10 h) and a slow pool (21 h). The half-life of the fast pool was not statistically different from that of lactose (but differed from that of fat and casein), while the half-life of the slow pool was statistically indistinguishable from milk fat and casein (but differed from lactose). The gross half-life was short for lactose (22 h) and longer for feces, whole milk, casein and milk fat (approx. 30 h).

The individual cows followed the same chase kinetics with the same parameter values as indicated by the mixed effects models. Also, no difference was apparent between the cows that grazed and those that were in stall immediately before the diet switch.

### Predicting Isotopic Fluctuations of Outputs

**Figure 3:**
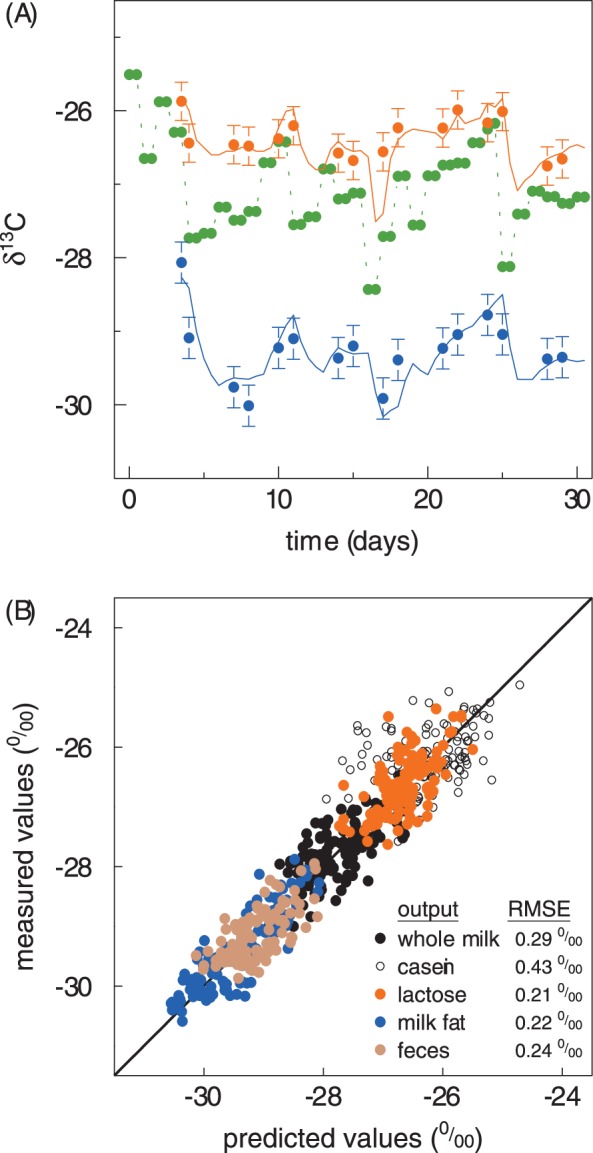
Validation of diet-switch based turnover information by forward modeling. Panel A shows fluctuations of δ^13^C in diet (green circles and dashed line) and of the products milk fat (blue circles) and lactose (orange circles) of different cows as obtained from measurements during the equilibration period and predictions by the compartmental model for milk fat and lactose (blue and orange line, respectively) obtained during the chase period. Error bars denote the 95% confidence intervals of the mean. Panel B compares all measured and predicted δ^13^C values of whole milk, milk components (casein, lactose and milk fat) and feces; n = 128 for each output. The 1∶1 line is represented by a solid line.

The dietary input δ^13^C varied randomly with a range of 2 ‰ during the equilibration period (Fig. 1). This variation was short-term and some variation of δ^13^C was also found in the outputs, but the variation there was attenuated and delayed. The output variation predicted from the input by forward modelling is shown, as an illustrative example, for milk fat and lactose of two different cows in Fig. 3A (n = 16 for each output and each animal). The modelled and the measured δ^13^C of the outputs for all animals kept in stall arrange around the 1∶1 line (Fig. 3B). None of the slopes differed statistically significantly from one, and none of the intercepts differed statistically from zero when the measured values of the outputs were separately regressed against their predicted values. The RMSEs were ∼ 0.2 ‰ (RMSE for whole milk 0.29 ‰, for lactose 0.21 ‰, for milk fat 0.22 ‰, and for feces 0.24 ‰) and within the confidence intervals of the measurements, except for casein, which exhibited a larger unexplained variation (RMSE 0.43 ‰). For the animals kept on pasture, this was essentially also true (RMSE increased by about 0.06 ‰ for all outputs, data not shown), when the unknown grass intake was replaced by the average grass intake of the respective cow as measured during its stall period and, the same δ^13^C was assumed for the grass intake as that measured for the same day for grass fed in stall.

## Discussion

Diet-switch based turnover models allowed the predicting of fluctuating isotopic compositions of metabolic outputs. This worked similarly well for outputs differing strongly in their genesis, namely milk, milk components and feces.

Despite their differing genesis, all outputs were kinetically rather similar. Delays, comprising ingestion, rumen and intestine passage, were short, but – although principally known (e.g., [42]) – to our knowledge not previously quantified and integrated into turnover models for lactating ruminants. As pointed out by Cerling et al. [9] neglecting the effects of these delays will cause erroneous turnover estimation, especially, when delay and half-life are similar in length. In our case, the gross half-life (delay plus metabolic half-life) thus overestimated the true metabolic half-life by about factor two (Table 1).

The turnover of all outputs was fast, especially compared to bovine muscle tissue (t_½_ ∼ 150 d, [12]). Nevertheless, significant differences among outputs were evident. The order of half-lives of milk components (lactose<casein<milk fat) was consistent with two other studies [43], [44], despite substantial differences between study designs (breed, diet, tracer injection site). The combination of short delays and short half-lives make milk and feces valuable non-destructive short-time indicators for food authentication and animal ecology that allow inferring the δ^13^C of the diet from 12 to 50 hours prior to milking (i.e. delay plus two half-lives, [24]).

Compartmental analysis identified two pools in milk that were not a combination of a fast and a slow pool supplying synthesis, but were due to the combination of three products differing in half-lives (with two being similar in half-life) as shown with the back calculated milk. An interpretation of multi-pool models is thus not straightforward in the case of heterogeneous products derived from different sources. Thus, this study also indicated that the statistical component separation cannot replace their analytical separation [45].

### Predicting Isotopic Fluctuations of Outputs

The good performance of the turnover models as derived from the chase period for predicting the isotopic variation during the equilibration period (Fig. 3) implies that all differences between both periods had little influence, namely the temporal type of isotopic variation (fluctuation vs switch), source of isotopic variation (grass vs maize), and ambient conditions (stall vs pasture), which potentially affect turnover (e.g., [19]). This good performance may be unexpected, especially because the diet differed between chase and equilibration period. We therefore conclude that the change in diet and the associated change in nutritional value were small enough not to confound the isotopic turnover. Two reasons to support this conclusion will be discussed: (i) the animals were kept at nutritional maintenance and thus milk and feces synthesis were almost entirely derived from exogenous sources, and (ii) the ruminal host-microbe interaction caused isotopic scrambling:

- At a level of nutrition below maintenance there is depletion of body stores, which then become an additional, endogenous source that enters the pool turnover for output synthesis [2]. If this were to occur it would need to be considered in the prediction. However, animal weights did not change. Therefore milk synthesis – within the detection limit of the study – was derived from the diet and pool turnover. This agrees with Boutton et al. [23] and Martin & Sauvant [46] who found that milk synthesis in mid and end lactation (for cows fed at maintenance, as herein) was almost exclusively derived from exogenous sources. The same should be the case for feces.
- Diet changes have been found to affect half-lives in non-ruminants [11], [20], [21]. In contrast, the ruminal host-microbe interaction converts dietary material into microbial biomass that is utilized [47]. This biomass is quite stable to diet switches as long as diet switches are small enough not to affect the ruminal or post-ruminal turnover [48], [49], which is likely to have been the case given the near-identical digestibility of the two rations [33].

The model validation was only possible because the equilibration period, which was intended to provide isotopically constant feed, showed a pronounced variation and that a record of the dietary input existed. It is beyond the scope of this study to identify the reasons for the fluctuations in the grass feed. However, ample possibilities for such fluctuations exist, as it is known that isotopic composition differs among species even of one photosynthetic type [7] and hence among parts of a grassland differing in species composition [50], but also with short-term fluctuations in water availability [51] and with ontogenesis [7], [52], among others. Hence it is likely that in studies where no continuous record of the dietary input existed, such fluctuations also occurred and obscured the turnover parameters. Even in the case of homogenized and manufactured feed, strong fluctuations may appear [53]. Therefore, continuous diet records are needed in isotope turnover studies or studies quantifying the isotopic shift [54]. This may even offer new approaches to turnover quantification that avoid the artificial diet switch.

### Different Isotopic Shifts between Outputs

The isotopic shifts caused by the diet switch were consistently higher in the milk outputs than in feces. The shift was largest in lactose. This indicated isotopic routing [55]. The starch from maize meal is known to be partly ruminally protected and can pass the rumen without degradation and incorporation into microbial biomass [56]. The ruminally protected starch is then enzymatically broken down to glucose in the small intestine, absorbed and largely metabolized to lactose. Hence, lactose exhibited a slightly larger shift (−2.9 ‰) than the shift in diet (−2.7 ‰) while the opposite was the case for the casein (−1.6 ‰), which is produced from the microbial biomass. The even smaller shift in feces likely was caused by the slightly lower digestibility of the grass than the maize [33] increasing the proportion of grass residues in the feces.

The discrepancies between the diet shift and the output shifts will also cause a variation of diet-to-output discrimination (“trophic shift”). This implies that these discriminations vary with diet type but even for the constant diet type during the equilibration period, the diet-to-output discrimination apparently varied due to the delay and attenuation of the isotopic signal as indicated by the fluctuating distance between the input and the outputs in Fig. 3A. Also discrimination between different outputs (e.g. between lactose and milk fat) will vary if delay and/or half-life vary between both outputs. Mathematically this must cause correlations between discrimination and the isotopic composition of the diet, which have frequently been found, but which are spurious [49]. The correlations do not indicate an influence of the isotopic composition itself, but indicate that the output is not in isotopic equilibrium with the input, which hinders the proper quantification of the input-output discrimination.

### Conclusions

Diet switch-based turnover models allowed a successful prediction of fluctuating isotopic output signatures at times beyond parameter estimation. This indicated that the diet change by omitting a small C_4_ component did not affect the turnover leading to milk, lactose, milk fat and feces. Milk was described best by a two-pool model, which was caused by the different half-lives of its components, while the contribution of different pools during synthesis could not be shown for any of the analysed milk components. The short half-lives caused the recent diet to dominate all products. A significant fluctuation during the intentionally constant equilibration period allowed the validation of the turnover model but indicated that continuous feed records are essential in studies quantifying turnover or trophic shifts.
